# Alteration of the lipid of red carp (*Cyprinus carpio*) during frozen storage

**DOI:** 10.1002/fsn3.971

**Published:** 2019-02-17

**Authors:** Noel Tenyang, Bernard Tiencheu, Fabrice Tonfack Djikeng, Azia Theresia Morfor, Hilaire Macaire Womeni

**Affiliations:** ^1^ Department of Biological Sciences, Faculty of Science University of Maroua Maroua Cameroon; ^2^ Department of Biochemistry, Faculty of Science University of Dschang Dschang Cameroon; ^3^ Department of Biochemistry, Faculty of Science University of Buea Buea Cameroon; ^4^ School of Agriculture and Natural Resources Catholic University Institute of Buea Buea Cameroon

**Keywords:** fatty acid composition, food analysis, frozen storage, FTIR, lipid oxidation, red carp

## Abstract

The aim of this study was to determine the oxidative stability of oil extracted from red carp fish frozen up to 9 months at −18°C. To assess oil stability of red carp fish, the analytical indexes and Fourier transform infrared (FTIR) spectroscopy were used. These methodologies used provided similar conclusions. Before frozen storage, the composition of fatty acids showed that red carp oil is a good source of polyunsaturated fatty acids (PUFAs) such as linoleic acid (C18:2ω‐6: 5.29% of total fatty acid), linolenic acid (C18:3ω3: 3.53% of total fatty acid), arachidonic acid (C20:4ω6: 3.68% of total fatty acid), eicosapentaenoic acid (C20:5ω‐3, EPA: 4,06% of total fatty acid), and docosahexaenoic acid (C22:6ω‐3: 3.02% of total fatty acid). During frozen storage, the free fatty acid and peroxide value increased, respectively, from 1.35% to 8.06% in oleic acid and 3.77 to 18.62 meq O_2_/kg in lipid, while the ratio of PUFA/SFA and polyene index decreased, respectively, from 0.58 to 0.25 and 0.30 to 0.09. The triglycerides also decreased with frozen duration. Therefore, for good fish quality, red carp fish must be stored for <3 months at −18°C.

## 1 INTRODUCTION

The freshness of fish is an important factor that contributes to the quality of fish (Bremner & Sakaguchi, 2000). Fish has very high nutritive value. Their lipids contain a high level of PUFA (Pasoz, Gallardo, Torres, & Medina, 2005). Depending upon the contents of important fatty acids, the importance of many fishes has been reported. Research studies have shown that the consumption of fish rich in PUFA such as eicosapentaenoic acid (EPA) and docosahexaenoic acid (DHA) has curative and preventive effects on cardiovascular diseases, cancer, hypertension, inflammation, fat glycemic control, and neurodevelopment in infants (Kris‐Etherton, Harris, & Appel, 2003; Schacky, 2000; Sidhu, 2003).

Freezing and frozen storage are important preservation methods that are largely used to retain fish dietary and nutritional value (Erickson, 1997). These methods are popular because the rate of spoilage through microbial, chemical, and enzymatic mechanisms is highly temperature dependent; thus, lowering the temperature significantly slows down these reactions and fish retains its freshness (Kolbe, Craven, Sylvia, & Morrissey, 2004). However, during frozen and freezing storage, lipids are subjected to some changes, mainly hydrolytic and autooxidative changes. These give rise to rancidity (off‐flavor) that strongly affects the shelf life of fish and their quality (Dragoev, Kiosev, Danchey, Ioncheya, & Genov, 1998; Richards & Hultin, 2002). The rate and extent of lipid autooxidation depends, among others, on the degree of fatty acid unsaturation, oxygen exposure, and storage time and temperature (Tomas & Aoon, 1990). .

The red carp are commercially important fatty fishes that are consumed in Cameroon. After catching and before eating, they were stored at low temperature to retain the quality of fish product.

Several works have reported on the effect of cold storage on the nutritional value of some fishes (Dragoev et al., 1998; Moini, Khoshkhoo, & Hemati Matin, 2012; Nazemroaya, Sahari, & Rezaei, 2011; Saeed & Howell, 2002; Tenyang, Womeni, Tiencheu, Villeneuve, & Linder, 2017). In all these works, the major damages occur during cold storage were hydrolysis and autooxidation. In spite of the studies that have already been conducted on fish, there is limited knowledge concerning the effect of frozen storage on the lipid quality of red carp consumed in Cameroon. Thus, the aim of this study was to evaluate the effect of frozen at −18°C on lipid oxidation parameters and fatty acid composition of red carp. The scientific information generated in this study would contribute toward determining the frozen condition that will retain most of lipid qualities and preserve polyunsaturated fatty acid of red carp fish.

## 2 MATERIALS AND METHODS

### 2.1 Chemicals

All chemicals and solvents used in this study were of analytical grade and were produced from SD‐fine Chemicals limited (Ahmedabad, India), HiMedia Laboratories Pvt. Ltd (Mumbai, India), and Sigma‐Aldrich (St. Louis, USA).

### 2.2 Fish sample collection and processing

Twenty red carp fish samples (200 ± 2.5 g average weight) were bought from Youpowe fish market in Douala (Littoral region of Cameroon). These fish were wild caught, and were caught from Wouri River, Cameroon. After being purchased, they were transported in iceboxes to the Laboratory of Food Biochemistry within 4 hr. In the laboratory, they were directly packaged in polyethylene bags and frozen at −18°C. Analysis of frozen fish was undertaken as fresh (0) and after 1, 3, 5 and 9 months under cold storage at −18°C.

### 2.3 Lipid extraction

The raw (unfrozen) and storage fish samples were eviscerated and washed with clean distilled water. They were then filleted, and lipids were extracted from fish samples by the method described by Bligh and Dyer (1959). The extracted oil was stored under refrigeration at 4°C for further analysis.

### 2.4 Effect of frozen storage on the quality of fish oil

The determination of free fatty acid (FFA) of fish oil samples was made according to the method described by AFNOR (1981). The results were expressed as % oleic acid. Its peroxide value (PV) was evaluated as described by Low and Ng (1978), and results expressed in meq O_2_/kg of lipid. Determinations were performed in triplicate.

### 2.5 Changes in fatty acid composition of red carp oil during frozen storage

Extraction of the total lipids was done using chloroform/methanol according to the method of Bligh and Dyer (1959). The lipids were transmethylated using NaOH/MeOH followed by BF_3_/MeOH according to the method described by Metcafe et al. (1966). The fatty acids methyl esters (FAMEs) were analyzed on a Hewlett Packard 5,880 gas chromatography (GC) equipped with a flame ionization detector. The esters were separated on a 50 m × 0.20 mm id wall‐coated open tubular fused‐silica capillary column coated with Carbowax 20 M. Column injector and detector temperature were 200 and 300°C, respectively. The carrier gas was helium, and the split ratio was 100/1. Identification of fatty acid was performed by comparison to retention times of authentic standards. All the experiments were carried out in triplicate.

### 2.6 Polyene index of fish oil (PI)

To determine lipid oxidation, the polyene index is a good indicator (Pirestani, Sahari, & Barzegar, 2010). The fatty acid ratio was calculated as follows: PI = C20:5 + C22:6/C16:0.

### 2.7 FTIR spectra analysis of red carp oil

IR spectra between 4,000 and 650 cm^−1^ were recorded using a perkin‐Elmer Spectrum 400 Infrared Spectrometer (Perkin‐Elmer Inc., Waltham, MA, USA) equipped with an ATR prism crystal accessory. The spectral resolution was 4 cm^−1^. Measurements were performed at room temperature using approximately 25 mg of extracted oil, which were placed on the surface of the ATR crystal, and pressed with a flat‐tip plunger until spectra with suitable peaks were obtained. The background was subtracted using the Spectrum software version 6.32 (Perkin‐Elmer Inc.).

### 2.8 Statistical analysis

Each determination was performed in triplicate. Data were expressed as mean ± standard derivation (*SD*). Statistics were performed using the Microsoft Office Excel program. Differences were evaluated by ANOVA using Statistical Package of Social Sciences (SPSS 16.0). Levels of statistical significance were *p* < 0.05.

## 3 RESULTS AND DISCUSSION

### 3.1 Chemical analyses of red carp oil

#### 3.1.1 Changes in free fatty acid

The free fatty acids (FFAs) of raw (unfrozen) and frozen red carp samples are presented in Table 1. The FFA is a common parameter in the specification of fats and oils. An increase in the level of FFA in fish oil or fat indicates hydrolysis of triglyceride by lipase and/or other factors like heat. The presence of high content of FFA in oil leads to the formation of off‐flavor as a result of rancidity of oil (Geromel & Montgomery, 1980). As shown in Table 1, FFAs of raw red carp sample and that stored for one month at −18°C are not significantly (*p* > 0.05) different. From the result, it was noted that the FFA of red carp oil tended to increase significantly (*p* < 0.05) after the first month of storage and sample stored for nine months had the highest value of FFA (8.06% oleic acid). Rodriguez et al. (2007), during frozen farmed coho salmon (*Oncorhynchus kisutch*), also observed the same trend in FFA. During frozen, triglycerides and phospholipids present in red carp fish undergo hydrolysis, resulting in the release of FFAs. These are due to the lysis of the cell membranes and the increase in some endogenous enzymes’ activities (Geromel & Montgomery, 1980). Kolakowska, Olley, and Dunstan (2002) found active phospholipase in fish pyloric caeca. Certain lipolytic enzymes can be produced by certain microorganisms. These enzymes increase the formation of FFA in fish during cold storage (Richards & Hultin, 2002). Bimbo (1998) proposed the maximum acceptable values of 5% FFA as per the quality specification for raw fish oil. This value was only reached after 3 months of storage of red carp at −18°C. Sikorski and Kolakowska (1994) reported that the formation of FFA itself does not lead to nutritional losses. However, accumulation of FFA has been related to some extent to lack of acceptability.

**Table 1:** Changes in acid and peroxide values of oils extracted from frozen red carp fish

	0 month	1 month	3 months	5 months	9 months
Acid value (% oleic acid)	1.35 ± 0.07^d^	1.97 ± 0.11^d^	4.07 ± 0.25^c^	5.77 ± 0.34^b^	8.06 ± 0.08^a^
Peroxide value (meq O_2_/kg lipid)	3.77 ± 0.71^d^	5.01 ± 0.33^d^	9.63 ± 0.69^c^	12.47 ± 0.20^b^	18.62 ± 0.53^a^

Note

Values are means ± standard error (*n* = 3). From triplicate determination, different letters in the same line indicate significant differences *p* < 0.05.

#### 3.1.2 Changes in Peroxide value (PV)

Lipid oxidation is a complex process induced by an initiation in combination with oxygen. To assess fish oil oxidation, measuring the level of hydroperoxide is commonly used. Hydroperoxide has little effect on the sensory properties of fish oil. Peroxide values of treated red carp were determined at 0, 1, 3, 5, and 9 months of storage at −18°C (Table 1). As shown in Table 1, untreated sample (0 month of storage) presented the lower PV (3.77 meq O_2_/kg of oil). The peroxide values of the untreated red carp (0 month) and that stored for one month do not differ significantly (*p* > 0.05).After the first month of storage at −18°C, the peroxide value of red carp oil increases significantly (*p* < 0.05) and red carp stored for nine months presented the highest value of PV (18.62 meq O_2_/kg of lipid). These trends are in accordance with those presented by Tenyang et al. (2017), when evaluating the effect of refrigeration on lipid oxidation of catfish. They are also in agreement with those presented by Saeed and Howell (2002), when studying the effect of frozen on oxidation of Atlantic mackerel. The increase in the amount of PV during frozen duration can be attributed to the higher formation of hydroperoxides due to the attack of unsaturated fatty acid present in red carp muscle. The PV of red carp fish stored for 9 months at −18°C was higher than those obtained by Rostamzad, Shabanpour, Shabani, and Shahiri (2011) on fillet of Persian fish after six months of frozen (10 meq O_2_/kg of lipid). Although literature review mentioned the peroxide value of crude oil between 3 and 20 meq O_2_/kg of lipid (Gokhan, Hikmet, & Muhammet, 2006), the value obtained here with red carp oil did not exceed 19 meq O_2_/kg of lipid, whereas the acceptable limit of peroxide value of crude oil is 7–8 meq O_2_/kg of lipid (Huss, 1988). This maximal suggested limit was reached in red carp fish oil only when they were frozen for more than three months. The red carp fish stored for 3 months were not rancid because the rancidity flavor occurs when peroxide value reach 20–40 meq O_2_/kg of lipid (Egan, Krik, & Sawyer, 1997).

#### 3.1.3 Changes in fatty acid composition

Fatty acid composition (% of total fatty acid) in fresh (untreated) and frozen samples of red carp fish is listed in Table 2. In fresh red carp, the amount of  saturated fatty acids (SFAs) was highest, while that of  polyunsaturated fatty acids (PUFAs) was lowest. These observations are in disagreement with those noted by Karami, Moradi, Motallebi, Hosseini, and Saltani (2013), which noted that in raw red tilapia, the amount of MUFAs far exceeded the SFAs content. Vlieg and Body (1998) showed that in freshwater fish, the content of PUFAs was lower, compared to that of marine fish. The difference can be attributed to fish food and species. Concerning the SFAs of fresh red carp (0 month) in this work, the predominant fatty acids were stearic acid (C18:0) and palmitic acid (C16:0), respectively. Lauric acid (C12:0) was the lowest SFA. Oleic acid (C18:1) and palmitoleic acid (C16:0) were the most important MUFAs present in red carp oil. Within the PUFAs, linoleic acid (C18:2) was the most important, followed by EPA (C20:5) and arachidonic acid (C20:4). EPA content in this study was higher than that of DHA. These findings are in disagreement with those obtained by Karami et al. (2013) in others oily fish. The total omega‐3 fatty acids (12.47%) in unfrozen red carp were higher compared to that of omega‐6 fatty acids (9.94%). These are in accordance with the results obtained by Tenyang et al. (2017) in raw herring. The high content of ω‐3 PUFAs and other PUFA in red carp increase the high quality of red carp oil from a cardiovascular point of view (Kris‐Etherton et al., 2003; Lindo & Hayakawa, 1996).

**Table 2:** Changes in fatty acid profile of red carp fish muscle during frozen

Fatty acids (g/100 g oil)	Storage time (months)				
	0	1	3	5	9
C12:0	0.18 ± 0.13^a^	0.14 ± 0.00^b^	0.19 ± 0.00^a^	0.18 ± 0.00^a^	0.21 ± 0.01^a^
C14:0	2.72 ± 0.13^b^	2.67 ± 0.01^b^	2.43 ± 0.20^b^	2.81 ± 0.04^b^	3.52 ± 0.03^a^
C16:0	23.69 ± 0.55^b^	23.46 ± 0.14^b^	23.63 ± 0.13^b^	25.60 ± 0.11^a^	26.37 ± 0.05^a^
C16:1	7.06 ± 0.03^d^	7.25 ± 0.15^d^	8.61 ± 0.06^c^	9.62 ± 0.08^b^	10.31 ± 0.01^a^
C17:0	2.11 ± 0.10^bd^	1.99 ± 0.11^b^	2.38 ± 0.01^ad^	2.34 ± 0.01^ad^	1.64 ± 0.02^c^
C17:1	1.55 ± 0.03^bc^	1.48 ± 0.02^c^	1.64 ± 0.06^b^	1.70 ± 0.01^ab^	1.81 ± 0.01^a^
C18:0	9.72 ± 0.08^c^	10.30 ± 0.25^bc^	11.08 ± 0.11^ab^	10.50 ± 0.19^bc^	11.32 ± 0.17^a^
C18:1n‐9	22.91 ± 0.08^d^	22.68 ± 0.04^d^	23.72 ± 0.03^c^	24.81 ± 0.10^b^	26.84 ± 0.21^a^
C18:2*n*‐6	5.29 ± 0.10^a^	5.15 ± 0.01^a^	4.65 ± 0.06^b^	4.11 ± 0.06^c^	3.43 ± 0.04^d^
C18:3*n*‐3	3.53 ± 0.12^a^	2.86 ± 0.04^b^	2.08 ± 0.05^c^	2.05 ± 0.04^c^	1.02 ± 0.03^d^
C20:1*n*‐9	1.44 ± 0.08^c^	1.62 ± 0.01^bc^	1.71 ± 0.07^bc^	1.92 ± 0.09^b^	2.60 ± 0.06^a^
C20:4*n*‐6	3.68 ± 0.13^a^	3.38 ± 0.06^ab^	3.33 ± 0.01^ab^	2.20 ± 0.08^c^	1.78 ± 0.06^a^
C20:5n‐3 (EPA)	4.06 ± 0.07^a^	4.01 ± 0.01^a^	3.56 ± 0.25^a^	2.72 ± 0.10^b^	2.02 ± 0.01^c^
C22:4*n*‐6	0.97 ± 0.01^a^	1.06 ± 0.00^a^	1.08 ± 0.08^a^	0.91 ± 0.01^a^	0.54 ± 0.01^b^
C22:5*n*‐3	1.86 ± 0.09^a^	1.53 ± 0.01^b^	1.27 ± 0.04^c^	0.84 ± 0.01^d^	0.31 ± 0.03^e^
C22:6*n*‐3 (DHA)	3.02 ± 0.10^ab^	2.88 ± 0.05^b^	2.64 ± 0.01^bc^	2.33 ± 0.11^c^	1.82 ± 0.10^d^
Saturated fatty acid (SFA)	38.42 ± 0.87^c^	38.56 ± 0.51^c^	39.71 ± 0.45^bc^	41.43 ± 0.44^ab^	43.06 ± 0.25^a^
Monounsaturated fatty acid (MUFA)	32.95 ± 0.25^c^	33.03 ± 0.23^c^	35.68 ± 0.24^b^	38.05 ± 0.28^b^	41.56 ± 0.29^a^
Polyunsaturated fatty acid (PUFA)	22.41 ± 0.66^a^	20.87 ± 0.18^a^	18.61 ± 0.49^b^	15.16 ± 0.41^c^	10.92 ± 0.28^d^
Σ*n*‐3	12.47	11.28	9.55	7.94	5.17
Σ*n*‐6	9.94	9.59	9.06	7.22	5.75
*n*‐3/*n*‐6	1.25	1.18	1.05	1.09	0.90
PUFA/SFA	0.58	0.54	0.47	0.36	0.25
PI	0.30	0.31	0.17	0.12	0.09
ΣNI	6.22	7.54	6.00	5.36	4.42

Note

NI = Unidentified fatty acid; values are means ± standard error (*n* = 3). From triplicate determination, different letters in the same line indicate significant differences *p* < 0.05.

Frozen storage at −18°C significantly changed the fatty acid profile of red carp (Table 2). As shown in Table 2, during storage, when storage time increased, total SFAs and MUFAs increased. The decrease in PUFAs especially EPA, DHA, and arachidonic acid was observed. The lowest values of these PUFAs (10.91%) were noted at 9 months of frozen. The significant decrease in PUFAs might be due to their susceptibility to oxidation. The ratios of *n*‐3/*n*‐6 and PUFA/SFA, which are indices widely used to evaluate the nutritional value of fat for human consumption are noted in Table 2. According to some nutritional recommendation (HMSO, 1994; Osman, Suriah, & Law, 2001), the PUFA/SFA ratio in human diet should not exceed 0.45, and within PUFA, *n*‐3/*n*‐6 ratio should not exceed 5. In the present experiment, the *n*‐3/*n*‐6 ratio decreases with frozen storage duration and frozen red carp for 9 months had the lowest ratio (0.90). All these samples had a recommended *n*‐3/*n*‐6 ratio. Concerning the PUFA/SFA ratio, the value decreases with frozen storage time and the lowest ratio (0.31) was obtained after 9 months of frozen at −18°C. The decrease in PUFA/SFA can be explained by the oxidation of PUFAs during storage, which would probably lead to the decrease in nutritional value of red carp. The PUFA/SFA ratio of the sample stored for less than three months was greater than the minimum permissive value of 0.45 as recommended by HMSO (1994).

Damage of PUFA during processing also was measured by PI value. PI might provide a meaningful tool to measure oxidative stability of fish oil. As shown in Table 2, PI decreases during frozen storage from 0.31 to 0.09, due to lipid oxidation, and samples stored at 9 months had the lowest value of PI. Similar observations were obtained by Sahari, Nazemroaya, and Rezaei (2009) in mackerel during storage. This study demonstrates that for good red carp, the frozen time should not exceed 3 months.

### 3.2 Evaluation of lipid oxidation during frozen storage of red carp fish by FTIR

**Figure 1:**
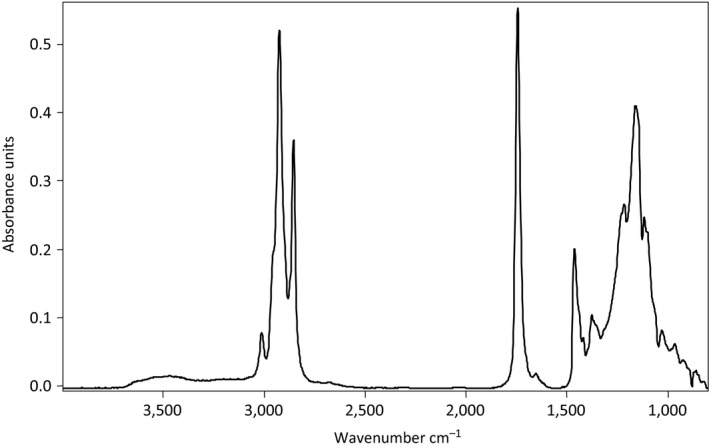
Representative Fourier transform infrared spectrum of lipids extracted from raw red carp fish in 3,800–800 cm^−1^ region

**Figure 2:**
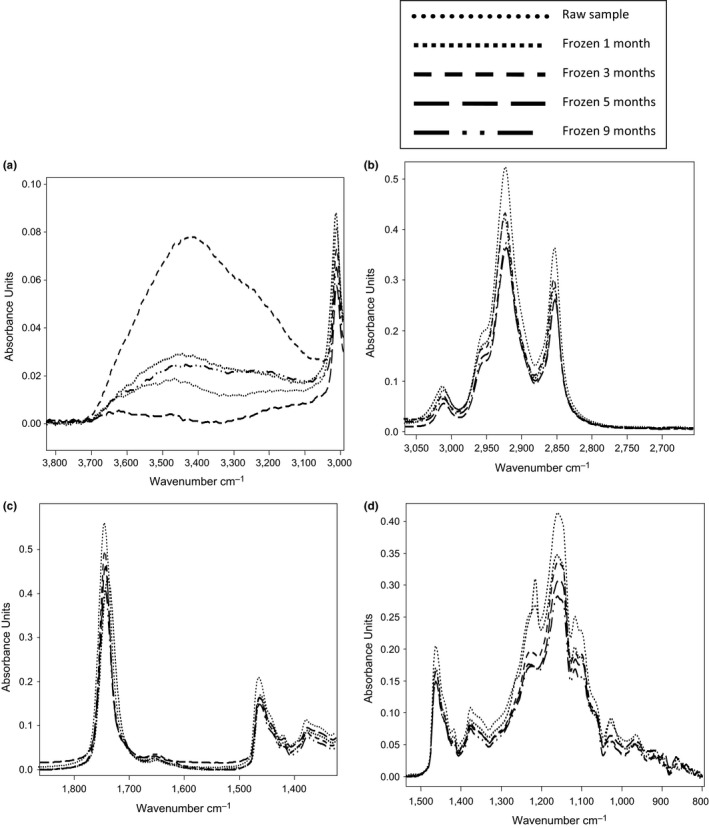
Selected regions (a–d) of FTIR spectra of lipid extracted from frozen red carp fish

Figure 1 shows the FTIR spectra of crude oil extracted from fresh red carp in the 3,800–800 cm^−1^ region. The FTIR spectrum exhibited similar regions of functional group vibration as reported previously by many workers (Manat, Soottawat, Wonnop, & Cameron, 2006; Vlachos et al., 2006). The spectrum of red carp oil as seen from Figure 1 is quite complex and contains several bands arising from the contribution of different functional groups of lipids. Below 1,800 cm^−1^, the major bands in the spectral region can be found at 1,250 cm^−1^ (stretching vibration of C‐O ester group), 1,150 cm^−1^ (bending vibration of the CH_2_ group), 1,655 cm^−1^ (stretching vibration of cis double bond, C = C), and 1,750 cm^−1^ (stretching vibration of C‐O ester group). The symmetric and asymmetric C‐H stretching vibrations of methyl and methylene groups appear respectively at around 2,853 and 2,925 cm^−1^. At about 3,010 cm^−1^, the symmetric C‐H stretching vibration link adjoining the double C = C link is observed. The region around 3,350 cm^−1^ (Figure 1) is assigned to the OH stretching vibration of hydroperoxides present in the oil. The detailed spectral analyses were performed in four distinct frequency ranges precisely 3,800–3,000, 3,000–2,800, 1,800–1,500, and 1,500–800 cm^−1^, respectively, as presented in Figure 2. In Figure 2a, the presence of the bands at the region between 3,800 and 3,100 cm^−1^ is linked to the hydroperoxide group present in the red carp fish. Any increase in the absorbance band during frozen indicates the formation of hydroperoxide. At the 5th month of frozen, the absorbance of this region decreases, suggesting the decomposition of hydroperoxide. Badui Dergal (2006) mentioned that hydroperoxides are intermediate metabolites during lipid oxidation of food, so their formation increases up to a maximum value and then start decreasing encouraging the production of aldehydes and ketones as final oxidation products. The increase in absorbance during the first three months of freezing was in agreement with the PV obtained by titrimetric method. In the same region, the band at 3,012 cm^−1^ could be a useful indicator of different degree of unsaturation in acyl chains of lipids (Guillén & Cabo, 1999). As seen in Figure 2a, around 3,012 cm^−1^, control sample and sample stored from one month presented the highest absorbance band, which means that they presented the higher unsaturation compared with other samples. However, the intensity of this band decreased with frozen duration. Frozen red carp for nine months recorded the lowest intensity of the band. The same conclusions were mentioned at 1,650 cm^−1^ wavenumber (Figure 2c). The decrease in the band at these wavenumbers was linked to the loss of unsaturation, and samples stored for nine months were more oxidized. These confirm the change noted in the region 3,800–3,100 cm^−1^. During frozen storage, the unsaturated acyl chains concentration present in fish lipid decreased, due to oxidation. Lipid oxidation of unsaturated fatty acids present in oil leads to the formation of free radicals, which can easily react with molecular oxygen and form hydroperoxides as primary oxidation products at the propagation stage (Pandey & Mishra, 2000). However, Guillén, Ruiz, and Cabo (2004) reported in salmon lipid that a reduction in the degree of lipid unsaturation due to eventual oxidative progress during storage time could not be evidenced by monitoring the peak at 3,012 cm^−1^. As indicated in Figure 2b, the bands at 2,950 and 2,852 cm^−1^ showed a reduction in absorbance during frozen storage and red carp stored for 5 and 9 months presented lesser intensity. These observations indicated an appreciable reduction of the concentration of the CH_3_ and CH_2_ functional groups present in the samples. Vlachos et al. (2006) noted that a reduction in the absorbance of these bands leads to the oxidative change observed in oils. Similar conclusion is noted at 1,460 and 1,370 cm^−1^ wavenumber (Figure 2d), respectively. Changes in the carbonyl absorption of the triglyceride ester linkage at around 1,750 cm^−1^ were reported as a main FTIR event denoting lipid oxidation. As shown in Figure 2c, at 1,750 cm^−1^, raw sample had the highest absorbance band and the decrease in the absorbance at this wavenumber was visible during frozen. Red carp stored for 9 months presented the least absorbance band. These decreases of this band at this wavenumber were associated with the decrease of triglyceride ester linkage due to eventual hydrolysis. Guillén et al. (2004) showed the change that occurs in this wavenumber is due to the formation of aldehydes and ketones, which are the secondary oxidative products derived from the degradation of the hydroperoxides. In conclusion, the samples stored for nine months were more affected during frozen. The bands associated with the fingerprint region at frequency values below 1,500 cm^−1^ showed a definite variation with respect to storage duration. The peaks at 1,280 and 1,050 cm^−1^ were more altered for the sample stored for 9 months (Figure 2d). The bands at 1,238 and 1,160 cm^−1^ associated with the stretching vibration of C‐O ester groups present in fish oil showed a clear tendency to decrease, particularly for the samples stored for 9 months, which had lesser absorbance band. The lower absorbance band obtained is the marker of more advanced oxidation. Vlachos et al. (2006) reported that the presence of the absorbance band at 967 cm^−1^ is associated with bending vibration of CH functional groups of isolated trans‐olefins. This band increases with advances oxidation linked to the production of trans double bonds in fatty acids in fish oil. In the case of this study, no difference seemed to exist between the samples.

## 4 CONCLUSION

Based on the data obtained from this study, before frozen storage, red carp lipid had the best quality. The red carp lipid is a good source of PUFAs such as EPA, DHA, and arachidonic acid, which are good for health. Frozen storage had an impact on the quality of red carp. The changes (decrease) observed in the proportion of PUFAs, PI, PUFA/SFA, and ω‐3/ω‐6 ratios indicated a substantial loss of nutritional quality of red carp during frozen storage. Therefore, red carp immediately frozen can be used up to 3 months because PVs obtained are within the acceptable limit of Codex Alimentarius (WHO/FAO).

## CONFLICT OF INTEREST

On behalf of all authors, the corresponding author states that there is no conflict of interest.

## ETHICAL STATEMENT

This article does not contain any studies with human participants or animals requiring ethical approval.

## References

[fsn3971-bib-0001] AFNOR (1981). Recueil des Normes Françaises. Corps Gras, Graines Oléagineuses, Produit Dérivés (2nd éd.), Paris, France:AFNOR, 438P.

[fsn3971-bib-0002] Badui Dergal,S. (2006). Quimica de los alimentos. cuarta edicion, Naucalpan, Mexico: Pearson Educacion, 736P.

[fsn3971-bib-0003] Bimbo,A. P. (1998). Guidelines for characterizing food‐grade fish oils. Vol. 9(5), Hertfordshire, UK: Informs.

[fsn3971-bib-0004] Bligh, E. C. , & Dyer, W. J. (1959). A rapid method of total lipid extraction and purification. Canadian Journal of Biochemistry and Physiology, 37, 911–917. 10.1139/o59-099 13671378

[fsn3971-bib-0005] Bremner, H. A. , & Sakaguchi, M. A. (2000). Critical look at whether freshness can be determined. Journal of Aquatic Food Product Technology, 9, 5–25. http://direct.bl.uk/bld/PlaceOrder.do?UIN=086451748&ETOC=RN&from=searchengine

[fsn3971-bib-0006] Dragoev, S. G. , Kiosev, D. D. , Danchey, S. A. , Ioncheya, N. I. , & Genov, N. S. (1998). Study on the oxidative processes in frozen fish. The Journal of Agricultural Science, 4(1), 55–65.

[fsn3971-bib-0007] Egan, H. , Krik, R. S. , & Sawyer, R. (1997). Pearson’s chemical analysis of food (pp.609–634) (9th Edn), Edinbugh, UK; Churchill Livingstone pp. 609-634.

[fsn3971-bib-0008] Erickson, M. (1997). Lipid oxidation: Flavor and nutritional quality deterioration in frozen foods In: Erickson M., & Hung Y. C. (Eds) Quality in frozen food (pp. 141–173). New York, NY: Chapman and Hall.

[fsn3971-bib-0009] Geromel, E. J. , & Montgomery, M. W. (1980). Lipase release from lysosomes of rainbow trout (*Salmo gairdnerii*). Journal of Food Science, 45(3), 412–419.

[fsn3971-bib-0010] Gokhan, B. , Hikmet, K. , & Muhammet, B. (2006). Changes in the quality of fish oils due to storage temperature and time. Food Chemistry, 98, 663–698. 10.1016/j.foodchem.2005.06.041

[fsn3971-bib-0011] Guillén, M. D. , & Cabo, N. (1999). Usefulness of the frequency data for the Fourier transform infrared spectra to evaluate the degree of oxidation of edible oils. Journal of Agriculture and Food Chemistry, 47, 709–719. 10.1021/jf9808123 10563958

[fsn3971-bib-0012] Guillén, M. D. , Ruiz, A. , & Cabo, N. (2004). Study of the oxidative degradation of farmed salmon lipids by jeans of fourier transforms infrared spectroscopy. influence of salting. Journal of the Science of Food and Agriculture, 84(12), 1528–1534. 10.1002/jsfa.1811

[fsn3971-bib-0013] HMSO (1994). Nutritional aspects of cardiovascular disease. Report on Health and Social Subject No. 46. London, UK: Department of Health. Her Majesty’s Stationery Office.

[fsn3971-bib-0014] Huss, H. H. (1988). Fresh fish quality changers. Rome, Italy: FAO.

[fsn3971-bib-0015] Karami, B. , Moradi, Y. , Motallebi, A. A. , Hosseini, E. , & Saltani, M. (2013). Effect of frozen on fatty acids profile, chemical quality indices and sensory properties of red tilapia (Oreochromis niloticus and tilapia mossambicus) fillets. Iranian Journal of Fisheries Sciences, 12(2), 378–388.

[fsn3971-bib-0016] Kaur, N. , Chugh, V. , & Gupta, A. (2014). Essential fatty acids as functional component of foods‐ A review. Journal of Food Science and Technology, 51(10), 2289–2303. 10.1007/s13197-012-0677-0 25328170 PMC4190204

[fsn3971-bib-0017] Kolakowska, A. , Olley, J. , & Dunstan, G. A. (2002). Fish lipids In: Sikorski Z. E., & Kolakowska A. (Eds.), Chemical and functional properties of food lipids (pp. 221–264). Boca Raton, FL: CRC Press.

[fsn3971-bib-0018] Kolbe, E. , Craven, C. , Sylvia, G. , & Morrissey, M. (2004). Chilling and freezing guidelines to maintain onboard quality and safety of albacore tuna agricultural experiment station. Astoria, OR: Oregon State University.

[fsn3971-bib-0019] Kris‐Etherton, P. M. , Harris, W. S. , & Appel, L. J. (2003). Omega‐3 fatty acids and cardiovascular disease: New recommendations from the American Heart Association. Arteriosclerosis, Thrombosis and Vascular Biology, 23, 151–152. 10.1161/01.atv.0000057393.97337.ae 12588750

[fsn3971-bib-0020] Lindo, Y. Y. , & Hayakawa, K. (1996). Docosahexaenoic acid: A valuable nutraceutical? Trends Food Science and Technology, 7, 59–62.

[fsn3971-bib-0021] Low, L. K. , & Ng, C. S. (1978). Determination of peroxide value In Hasegawa H. (Ed.), Laboratory manual on analytical methods and procedures for fish and fish products (pp. C7.1–C7.3). Singapore, Singapore: Marine Fisheries Research Department, Southeast Asian Fisheries Development Center.

[fsn3971-bib-0022] Manat, C. , Soottawat, B. , Wonnop, V. , & Cameron, F. (2006). Changes of lipids in sardine (*Sardinella gibbosa*) muscle during ices storage. Food Chemistry, 99, 83–91. 10.1016/j.foodchem.2005.07.022

[fsn3971-bib-0023] Metcalfe, L. D. , Schmitz, A. A. , & Pelka, J. R. (1966). BF3‐methanol procedure for rapid quantitative preparation of methyl esters from lipids. Analytical Chemistry, 38, 514.

[fsn3971-bib-0024] Moini, S. , Khoshkhoo, Z. H. , & Hemati Matin, R. (2012). The fatty acids profiles in mackerel (Scomberomorus guttatus) and its shelf life in cold storage at ‐18 °C. Global Veterinaria, 8(6): 665–669.

[fsn3971-bib-0025] Nazemroaya, S. , Sahari, M. A. , & Rezaei, M. (2011). Identification of fatty acid in mackerel (*Scomberomorus commersoni*) and shark (*Carcharhinus dussumieri*) fillets and their changes during six month of frozen at ‐18 °C. Journal of Agricultural Science and Technology, 13, 553–556.

[fsn3971-bib-0026] Osman, H. , Suriah, A. R. , & Law, E. C. (2001). Fatty composition and cholesterol content of selected marine fish in Malaysian waters. Food Chemistry, 73, 55–60. 10.1016/s0308-8146(00)00277-6

[fsn3971-bib-0027] Pandey, B. N. , & Mishra, K. P. (2000). Fluorescence and ESR studies on membrane oxidative damage by gamma‐radiation. Applied Magnetic Resonance, 18, 483–492. 10.1007/bf03162295

[fsn3971-bib-0028] Pasoz, M. , Gallardo, J. M. , Torres, J. L. , & Medina, I. (2005). Activity of grape polyphones as inhibitors of the oxidation of fish lipids and frozen fish muscle. Food Chemistry, 92, 547–557. 10.1016/j.foodchem.2004.07.036

[fsn3971-bib-0029] Pirestani, S. , Sahari, A. , & Barzegar, M. (2010). Fatty acids changes during frozen storage in several fish species from South Caspian Sea. Journal of Agricultural Science and Technology, 12, 321–329.

[fsn3971-bib-0030] Richards, M. , & Hultin, H. (2002). Contributions of blood components to lipid oxidation in fish muscle. Journal of Agriculture and Food Chemistry, 50, 555–564. 10.1021/jf010562h 11804529

[fsn3971-bib-0031] Rodriguez, A. , Losada, V. , Larrain, M. A. , Quitral, V. , Vinagre, J. , & Aubourg, S. P. (2007). Development of lipid changes related to quality loss during the frozen storage of farmed Coho Salmon (*Oncorhynchus kisutch*). Journal of the American Oil Chemists’ Society, 84(8), 727–734. 10.1007/s11746-007-1098-5

[fsn3971-bib-0032] Rostamzad, H. , Shabanpour, B. , Shabani, A. , & Shahiri, H. (2011). Enhancement of the storage quality of frozen Persian sturgeon fillets by using of ascorbic acid. International Food Research Journal, 18, 109–116.

[fsn3971-bib-0033] Saeed, S. , & Howell, N. (2002). Effect of lipid oxidation and frozen storage on muscle proteins of atlantic mackerel (*scomber scombrus*). Journal of the Science of Food and Agriculture, 82, 579–586. 10.1002/jsfa.1080

[fsn3971-bib-0034] Sahari, M. A. , Nazemroaya, S. , & Rezaei, M. (2009). Fatty acid and biochemical changes in Mackerel (Scomberomorus commerson) and Shark (*Carcharhinus dussumieri*) fillets during frozen storage. American‐Eurasian Journal of Sustainable Agriculture, 3(3), 519–527.

[fsn3971-bib-0035] Schacky, C. V. (2000). n‐3 fatty acids and the prevention of coronary atherosclerosis. The American Journal of Clinical Nutrition, 71, 224–227. 10.1093/ajcn/71.1.224s 10617975

[fsn3971-bib-0036] Sidhu, K. S. (2003). Health benefits and potential risks related to consumption of fish or fish oil. Regulatory Toxicology and Pharmacology, 38, 336–344. 10.1016/j.yrtph.2003.07.002 14623484

[fsn3971-bib-0037] Sikorski, Z. , Kolakowska, A . (1994). Changes in protein in frozen stored fish. In. Sikorski Z, Sun Pan B and Shahidi F, Sea food proteins (pp. 99–112). New York, NY: Chapman and Hall.

[fsn3971-bib-0038] Tenyang, N. , Womeni, H. M. , Tiencheu, B. , Villeneuve, P. , & Linder, M. (2017). Effect of refrigeration time on the lipid oxidation and fatty acid profiles of catfish (Arius maculatus) commercialized in Cameroon. Grasas Y Aceites, 68(1): 1371–9.

[fsn3971-bib-0039] Tomas, M. C. , & Aoon, M. C. (1990). Study on the influence of freezing rate on lipid oxidation in fish (salmon) and chicken breast muscles. International Journal of Food Technology, 25, 718–721. 10.1111/j.1365-2621.1990.tb01134.x

[fsn3971-bib-0040] Vlachos, N. , Skopelitis, Y. , Psaroudaki, M. , Konstantinidou, V. , Chatzilazarou, A. , & Tegou, E. (2006). Applications of Fourier transform‐infrared spectroscopy to edible oils. Analytical Chemistry Acta, 573, 459–465. 10.1016/j.aca.2006.05.034 17723561

[fsn3971-bib-0041] Vlieg, P. , & Body, D. R. (1998). Lipid contents and fatty acid composition of some new Zealand freshwater finfish and marine finfish shellfish, and roes. New Zealand Journal of Marine and Freshwater Research, 22, 151–162. 10.1080/00288330.1988.9516287

